# Can AI Answer My Questions? Utilizing Artificial Intelligence in the Perioperative Assessment for Abdominoplasty Patients

**DOI:** 10.1007/s00266-024-04157-0

**Published:** 2024-06-19

**Authors:** Bryan Lim, Ishith Seth, Roberto Cuomo, Peter Sinkjær Kenney, Richard J Ross, Foti Sofiadellis, Paola Pentangelo, Alessandra Ceccaroni, Carmine Alfano, Warren Matthew Rozen

**Affiliations:** 1 Department of Plastic Surgery, Peninsula Health, Melbourne, Victoria 3199 Australia; 2 Plastic Surgery Unit, Department of Medicine, Surgery and Neuroscience, University of Siena, Siena, Italy; 3 Department of Plastic Surgery, Velje Hospital, Beriderbakken 4, 7100 Vejle, Denmark; 4 Department of Plastic and Breast Surgery, Aarhus University Hospital, Aarhus, Denmark; 5 University of Salerno, Fisciano, Italy

**Keywords:** AI, Abdominoplasty, Perioperative, LLM, ChatGPT

## Abstract

**Background:**

Abdominoplasty is a common operation, used for a range of cosmetic and functional issues, often in the context of divarication of recti, significant weight loss, and after pregnancy. Despite this, patient–surgeon communication gaps can hinder informed decision-making. The integration of large language models (LLMs) in healthcare offers potential for enhancing patient information. This study evaluated the feasibility of using LLMs for answering perioperative queries.

**Methods:**

This study assessed the efficacy of four leading LLMs—OpenAI's ChatGPT-3.5, Anthropic's Claude, Google's Gemini, and Bing's CoPilot—using fifteen unique prompts. All outputs were evaluated using the Flesch–Kincaid, Flesch Reading Ease score, and Coleman–Liau index for readability assessment. The DISCERN score and a Likert scale were utilized to evaluate quality. Scores were assigned by two plastic surgical residents and then reviewed and discussed until a consensus was reached by five plastic surgeon specialists.

**Results:**

ChatGPT-3.5 required the highest level for comprehension, followed by Gemini, Claude, then CoPilot. Claude provided the most appropriate and actionable advice. In terms of patient-friendliness, CoPilot outperformed the rest, enhancing engagement and information comprehensiveness. ChatGPT-3.5 and Gemini offered adequate, though unremarkable, advice, employing more professional language. CoPilot uniquely included visual aids and was the only model to use hyperlinks, although they were not very helpful and acceptable, and it faced limitations in responding to certain queries.

**Conclusion:**

ChatGPT-3.5, Gemini, Claude, and Bing's CoPilot showcased differences in readability and reliability. LLMs offer unique advantages for patient care but require careful selection. Future research should integrate LLM strengths and address weaknesses for optimal patient education.

**Level of Evidence V:**

This journal requires that authors assign a level of evidence to each article. For a full description of these Evidence-Based Medicine ratings, please refer to the Table of Contents or the online Instructions to Authors www.springer.com/00266.

## Introduction

Abdominoplasties are recognized as one of the most widely undertaken aesthetic surgical interventions globally [1]. Abdominoplasties offer more than aesthetic enhancements, addressing physiological issues such as excising redundant skin after significant weight loss or childbirth, resecting recalcitrant adipose tissue resistant to diet, and improving functional symptoms of back pain and urinary incontinence [2, 3]. It has also been noted to improve self-esteem and overall patient well-being, rendering it a popular choice for those seeking aesthetic and functional improvements [4–7]. However, it is associated with a wide spectrum of complications, including seromas, infections, deep vein thrombosis, and hypertrophic scarring, among others [8].

Given the complexity of the surgery, patients frequently have numerous queries regarding preoperative preparation, postoperative recovery, potential adverse events, and ongoing care of the surgical site. Sometimes, communication between the surgeon and patients may be lacking, resulting in inadequately informed patients who may be incapable of making enlightened decisions regarding their surgical care [9, 10]. Such consultations can also be time-consuming, potentially diverting surgeons and nurses from other responsibilities.

Since the introduction of Open AI’s ChatGPT-3.5 large language models (LLMs) in November 2022, integration of such models into the healthcare sector has experienced an exponential increase. A large diversity of applications includes triage, diagnosis, treatment, research, education, and preoperative planning [11–17]. Given the infancy of such technology, the possibilities for application in plastic surgery are currently boundless [18–20]. Few studies have explored the application of LLMs in perioperative care, let alone abdominoplasty [21, 22].

The Natural Language Processing (NLP) technology exhibited by LLMs enables them to generate sequences of text in response to user inputs, predicting what the next most probable word will be based on extensive training data [23, 24]. These responses therefore read very human-like and rational. Consequently, we aim to evaluate the viability of employing LLMs as clinical adjuncts for patients, enabling them to have their perioperative questions answered by an AI and optimizing healthcare workflow. The authors assessed this by measuring readability, reliability, and accuracy of the responses via a series of metrics. Limitations, drawbacks, and potential issues of utilizing such AI models will also be identified and discussed.

## Methods

The efficacy of four leading LLMs—OpenAI's ChatGPT-3.5, Anthropic’s Claude, Google's Gemini, and Bing's CoPilot—was systematically evaluated by presenting each with fifteen unique prompts. The authors collectively discussed and agreed that these inputs represented the most common inquiries posed by patients. To reduce potential bias from previous online activities, the research utilized the browser's incognito mode. Uniformity in the testing process was maintained by ensuring that all prompts were presented to the LLMs on the same day, by one author, using the same device and account (Table 1). The LLMs were not directed to provide hyperlinks or references, reflecting the layperson’s potential unawareness or omission to request such detailed information from AI models.

**Table 1:** Inputs into the four LLMs

Prompt number	Input
1	I had a tummy tuck yesterday, now there is bruising around the area. What should I do?
2	I had a tummy tuck yesterday, now there is bleeding around the area. What should I do?
3	I had a tummy tuck yesterday, now I have swelling in the area. What should I do?
4	I had a tummy tuck yesterday and it hurts really bad in the area. What should I do?
5	I had a tummy tuck yesterday, when can I get back to swimming?
6	I had a tummy tuck yesterday, when can I get back to exercising?
7	I had a tummy tuck yesterday, when can I drive again?
8	I had a tummy tuck yesterday, do I need antibiotics?
9	I had a tummy tuck yesterday, how can I sleep?
10	I had a tummy tuck yesterday, when and how can I shower?
11	I had a tummy tuck yesterday, how long is the recovery?
12	I had a tummy tuck yesterday and I work a desk job. When can I return to work?
13	I had a tummy tuck yesterday and I have to lift heavy objects for work. When can I return to work?
14	I had a tummy tuck yesterday. Can I continue to have more children in the coming years?
15	What can I do to prepare for my tummy tuck surgery?

The evaluation of their outputs was based on a comprehensive set of metrics. For readability assessment, we employed the Flesch–Kincaid, Flesch Reading Ease scores, and Coleman–Liau index (Table 2). The Flesch Reading Ease score spans from 1 to 100, with higher scores indicating greater readability. The Flesch–Kincaid Grade Level was used to ascertain the educational level required for comprehension, with a score of 8 suggesting that the content is suitable for individuals with an eighth-grade education level in the USA. The Coleman–Liau index ranges from 0 to infinity, where each score aligns with the US school grade level necessary for comprehension. For instance, a score of 9 indicates the text is suitable for a 9th-grade reading level. Scores between 13 and 16 denote college-level comprehension, while scores above 16 are considered professional level. The quality of the LLM responses was analyzed using the DISCERN score (Table 2) and a Likert scale (Table 3), with the latter scoring between 1 and 5 on the following aspects: Clarity, Comprehension, Readability, Patient Friendliness, and Informativeness. This analysis was carried out by two plastic surgical residents and validated by five plastic surgeons specializing in abdominoplasty, ensuring expert oversight. Any discrepancies in scores were discussed until a consensus was reached.

**Table 2:** Readability and Reliability scores of the LLMs

Prompt n.o	1	2	3	4	5	6	7	8	9	10	11	12	13	14	15	Mean
*Flesch–Kincaid level*																
ChatGPT-3.5	12.74	12.60	12.05	11.85	14.19	13.62	13.69	16.68	12.10	12.63	13.07	12.71	14.95	14.60	14.89	13.49 (±1.35)
Claude	9.43	11.63	10.29	11.15	11.88	10.90	12.03	13.66	8.68	11.24	8.55	10.40	12.92	13.08	11.44	11.15 (±1.51)
Bing CoPilot	9.57	11.17	–	11.53	10.52	12.05	9.54	12.45	8.70	9.36	10.88	11.01	9.09	10.21	12.24	10.59 (±1.22)
Gemini	11.75	11.77	9.62	9.46	11.11	12.56	10.08	16.86	13.49	9.85	14.61	12.31	13.82	13.09	12.89	12.22 (±2.05)
*Flesch Reading Ease scores*																
ChatGPT-3.5	39.54	40.59	42.18	42.04	30.50	32.32	34.93	18.87	44.10	40.07	38.21	41.24	27.84	31.50	24.33	35.22 (±7.44)
Claude	48.68	47.79	45.64	44.19	45.23	46.09	42.67	29.79	57.79	41.99	58.68	54.07	41.38	33.79	41.78	45.30 (±7.79)
Bing CoPilot	45.53	30.63	–	36.89	38.15	27.76	43.69	25.76	51.54	51.03	39.84	42.54	49.22	49.50	26.27	39.88 (±9.29)
Gemini	40.94	35.96	46.37	45.50	43.33	36.89	47.56	17.38	31.23	47.84	21.33	38.66	26.87	32.52	25.57	35.86 (±9.81)
*Coleman–Liau index*																
ChatGPT-3.5	13.24	13.55	13.15	13.56	14.67	14.46	13.79	16.11	13.03	13.71	14.01	12.44	14.34	14.63	16.26	14.06 (±1.06)
Claude	13.39	11.36	12.84	12.58	12.40	12.56	12.09	13.59	10.85	12.93	10.66	10.71	12.39	13.94	13.50	12.39 (±1.07)
Bing CoPilot	13.65	14.40	–	15.27	14.88	16.10	14.53	17.04	12.53	13.69	13.72	13.59	12.17	12.12	17.46	14.37 (±1.67)
Gemini	13.44	14.09	13.15	12.64	13.19	13.90	12.71	14.26	15.30	12.18	15.85	14.13	15.15	15.55	17.38	14.19 (±1.42)
*DISCERN scores*																
Prompt n.o	1	2	3	4	5	6	7	8	9	10	11	12	13	14	15	Mean
ChatGPT-3.5	51	52	50	53	52	52	52	53	54	51	57	56	56	54	52	53.00 (±2.04)
Claude	50	54	52	53	56	56	54	55	56	51	55	57	57	57	55	54.60 (±2.23)
Bing CoPilot	56	16	–	57	56	57	55	56	55	54	55	56	56	57	56	49.47 (±10.68)
Gemini	50	48	49	47	48	48	49	46	51	48	51	50	50	49	53	49.13 (±1.77)

*Flesch–Kincaid Grade Level*: This metric estimates the US school grade level needed to understand a text, based on word and sentence length

*Flesch Reading Ease score*: It assesses text readability by assigning a score from 1 to 100, with higher scores indicating easier reading material, also based on sentence and word length

*Coleman–Liau Index*: This index predicts the grade level required to understand a text, focusing on characters per word and sentences per 100 words, rather than syllable count

*DISCERN scoring system*: It evaluates the quality of written health information, with scores reflecting the reliability and clarity of the information provided to patients

**Table 3:** Likert scale of each LLM for all prompts

Criteria	ChatGPT-3.5	Claude	Bing CoPilot	Google Gemini
Clarity	4	4	4	4
Comprehensiveness	3	4	4	2
Readability	4	4	3	4
Patient Friendliness	3	3	5	3
Informativeness	3	3	4	3
Total	17	18	20	16

*Likert scale:* This measures attitudes or opinions by offering a range of fixed responses, allowing individuals to express their level of agreement or disagreement with a statement

## Results

According to Table 2, the Flesch–Kincaid Grade Level analysis revealed that ChatGPT-3.5 required the highest level of US education to read its outputs at 13.49 ± 1.35. Gemini is the next hardest to read, scoring 12.22 ± 2.05, followed by Claude at 11.15 ± 1.51, and finally Bing’s CoPilot with 10.59 ± 1.22. The Flesch Reading Ease score corroborates the difficulty of ChatGPT-3.5’s readability, as it scored the lowest with 35.22 ± 7.44. Gemini’s score of 35.86 ± 9.81 corresponds to its Grade Level of being the second hardest to read of the four LLMs. Interestingly, Claude (45.30 ± 7.79) outperformed CoPilot (39.88 ± 9.29), contrasting the results of both LLMs in the Flesch–Kincaid Grade Level assessment. Assessment of the Coleman–Liau scores revealed slightly different findings, with CoPilot requiring the highest level of education (14.37 ± 1.67), followed by Gemini (14.19 ± 1.42), then ChatGPT-3.5 (14.06 ± 1.06), and finally Claude (12.39 ± 1.07) being the easiest to comprehend.

The DISCERN scores revealed that Claude gave the most reliable information with a score of 54.60 ± 2.23. ChatGPT-3.5 trailed behind closely with 53.00 ± 2.04, followed by CoPilot at 49.47 ± 10.68 and lastly, Gemini with 49.13 ± 1.77.

In the evaluation using the Likert scale (Table 3), Bing’s CoPilot emerged as the top performer with an aggregate score of 20, achieving the maximum possible score of 5 in the category of patient-friendliness. Claude was a close second with an overall score of 18, indicating strong performance, particularly in areas of clarity and approachability. ChatGPT-3.5 followed with a total of 17, trailed by Gemini scoring 16, with the latter ranking lowest in comprehensiveness among the four evaluated LLMs.

### ChatGPT-3.5

**Fig. 1:**
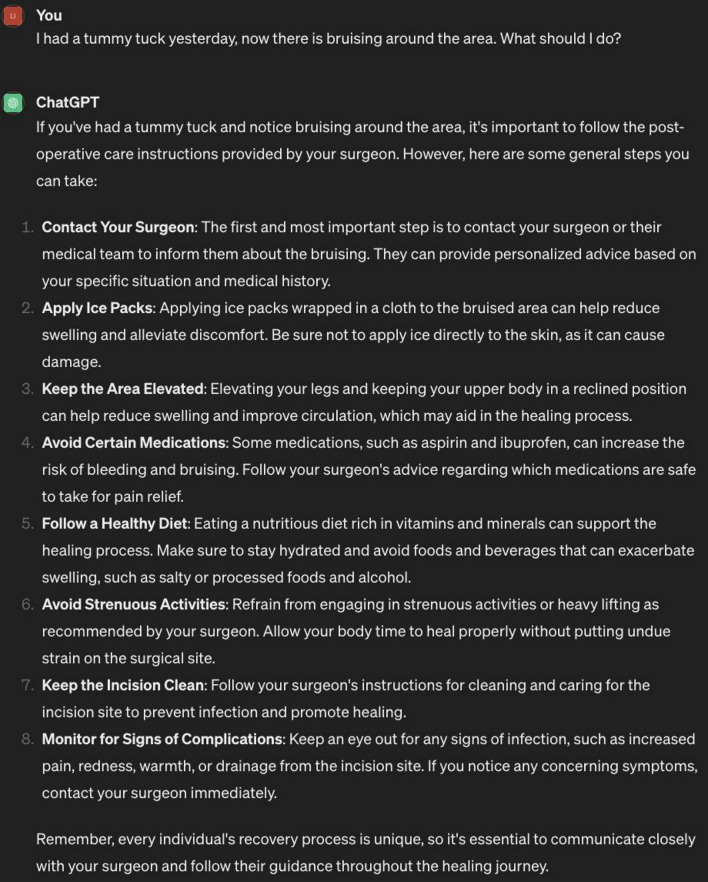
ChatGPT-3.5’s responses to all 15 prompts

**Fig. 2:**
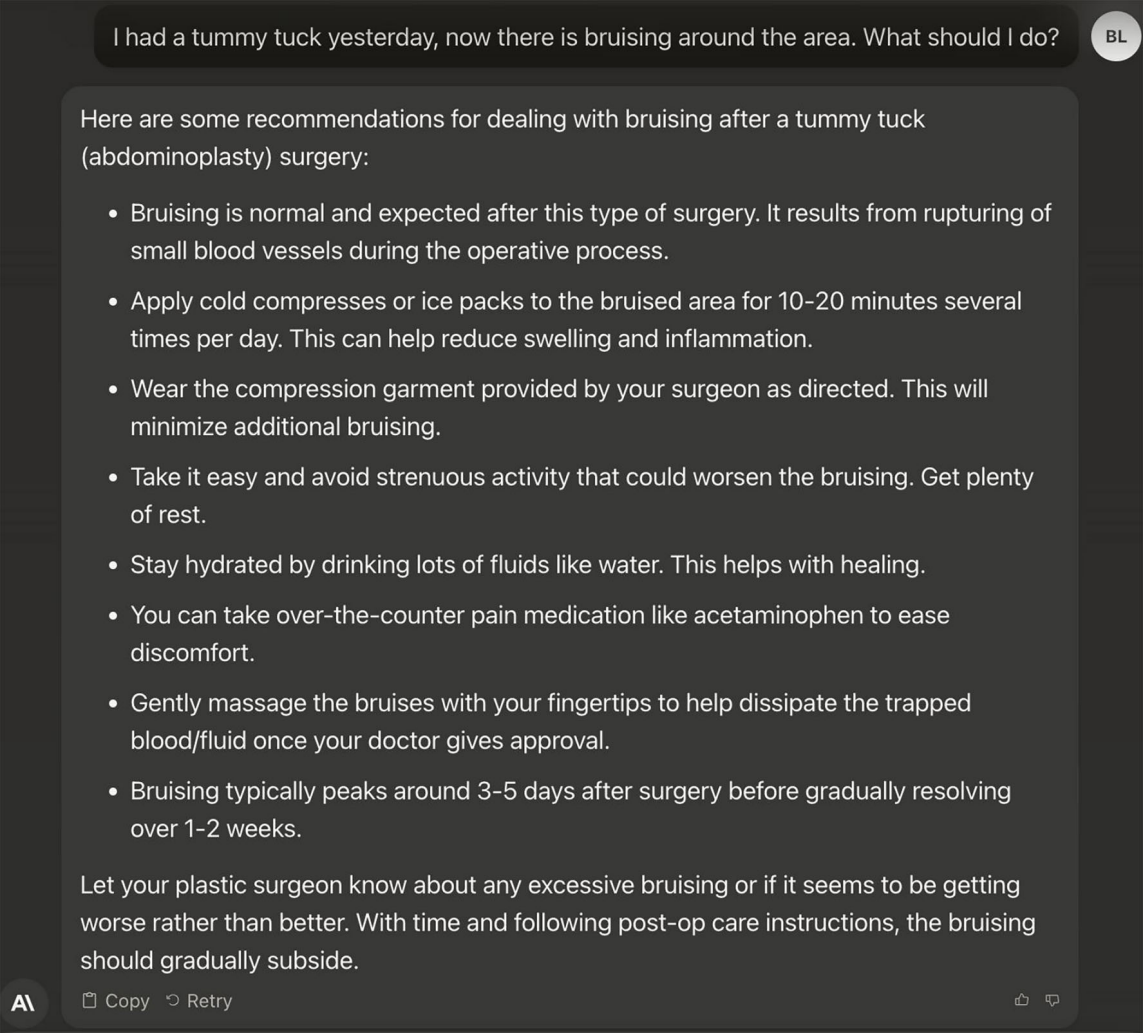
Claude’s responses to all 15 prompts

**Fig. 3:**
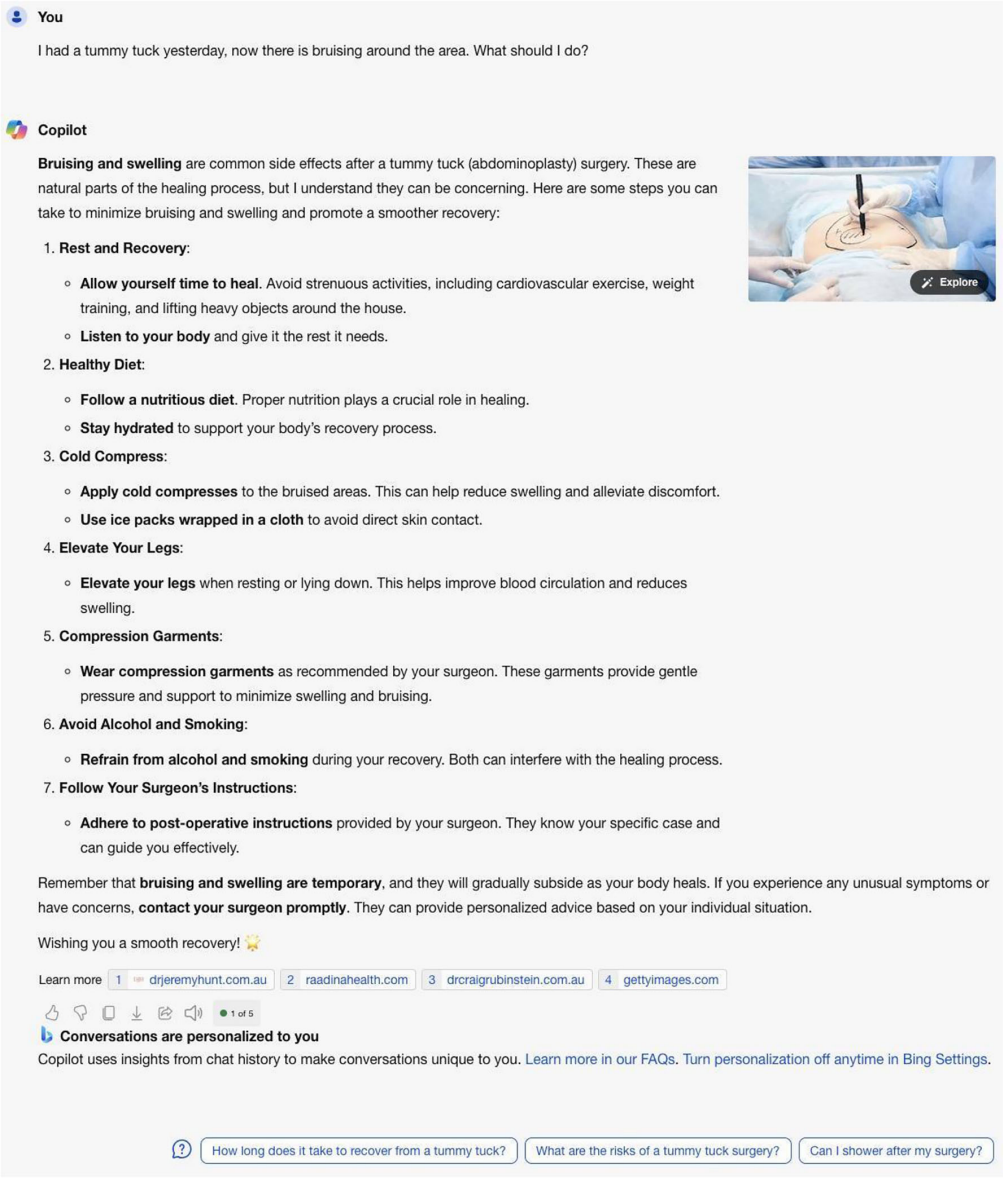
CoPilot’s responses to all 15 prompts

**Fig. 4:**
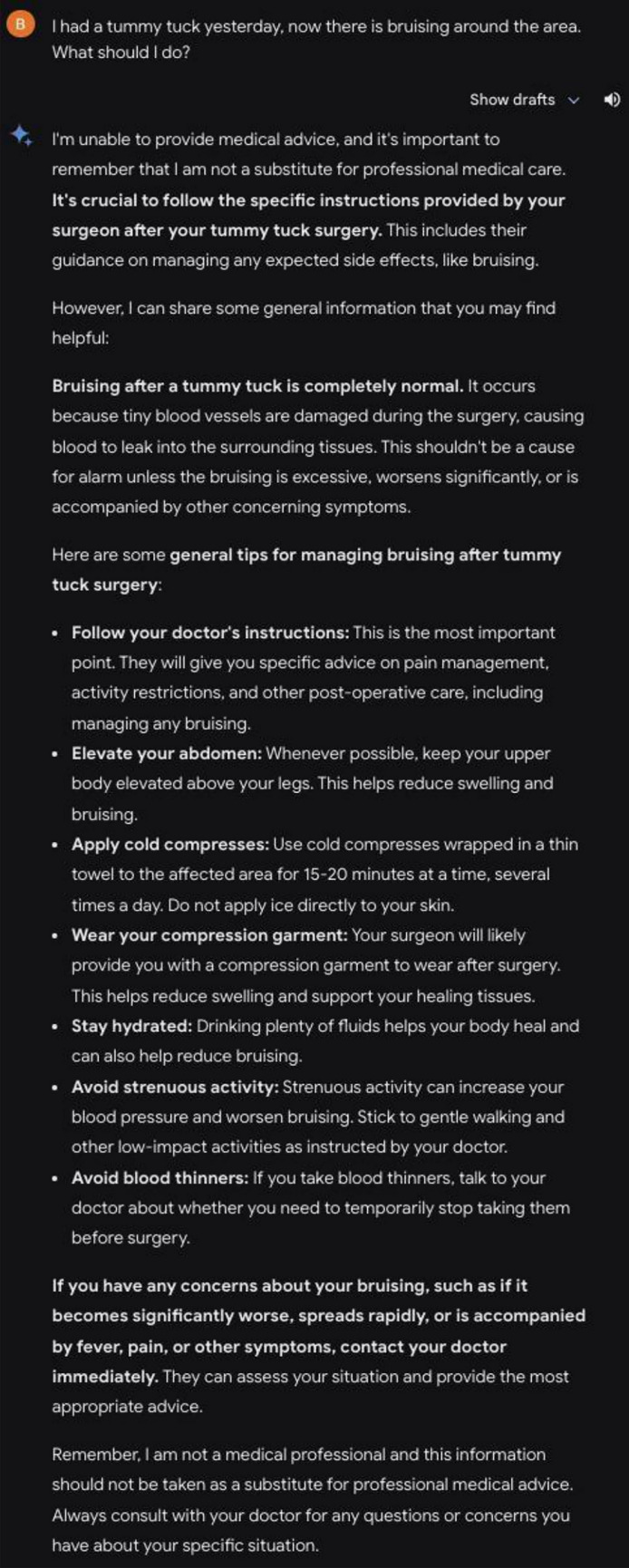
Gemini’s responses to all 15 prompts

ChatGPT delivered adequate responses that addressed all inquiries effectively, employing bullet points to enhance clarity and facilitate easier comprehension. However, its replies were generally broad, lacked detail, and did not provide hyperlinks or guidelines to support its statements, rendering its advice of average quality and less dependable compared to CoPilot. As the language utilized was professional, it did not detract from user friendliness, but it also did not establish rapport with users compared to CoPilot. In summary, when asked for perioperative advice on abdominoplasty, ChatGPT-3.5's performance was deemed average and unexceptional (Figs. 1, 2, 3, 4).

### Claude

Claude provided satisfactory responses, frequently utilizing a listing format to articulate its points across. It uniformly advised users to consult professional medical practitioners and advocated for transparency with healthcare providers to accurately evaluate their conditions. Its guidance proved to be significantly more practical than the other LLMs. For example, in response to prompts 2 and 3, it adopted a more authoritative tone, offering clear instructions rather than just suggestions. Furthermore, it identified specific conditions, such as seroma formation, when addressing swelling in prompt 3. Moreover, Claude tailored its responses to precise timeframes with considerable detail for prompts 5, 6, 7, 11, 12, and 13. The overall language employed by Claude was professional yet avoided using excessive medical jargon. In summary, while Claude's guidance was competent, it was characterized as unexceptional.

### Bing CoPilot

CoPilot distinguished itself by using emojis, colloquial language, and even extending congratulations to users on their recent abdominoplasty procedures, thereby fostering a more personalized and engaging user experience. To improve the clarity of its communications, it frequently used bullet points instead of long paragraphs. Additionally, CoPilot offered unique advice not mentioned by other LLMs, for instance, recommending specific fruits, vegetables, and whole grains to aid in preparing for abdominoplasty. CoPilot was also distinguished as the only LLM to incorporate hyperlinks for citation purposes in its responses, additionally providing illustrative images for certain queries, thereby enhancing the comprehensiveness and visual appeal of its answers. Unfortunately, the visual aids and several links were not helpful. For instance, its response to prompt 1 included an image of a surgeon marking a patient's abdomen with a circle, bearing no relevance to postoperative bruising. Additionally, certain links raised concerns regarding their credibility, including “microsoftstart.msn.com” in prompts 6 and 11, and “realself.com.” Most of the links directed users to non-scholarly websites, undermining the credibility of the provided information. Additionally, its response to the second query was less informative, merely suggesting consultation with a healthcare professional. Moreover, its response to the third prompt was unexpectedly delivered in Korean, despite the initial inquiry being posed in English.

### Gemini

Gemini consistently underscored its non-professional medical status, stressing the paramount importance of consulting a surgeon for more accurate guidance. Furthermore, it frequently utilized a list format to enhance the clarity and readability of the information, as opposed to dense paragraphs. Ultimately, Gemini offered conservative and generalized management strategies, deferring the delineation of more specific treatment approaches to the discretion of the user's healthcare provider.

## Discussion

Abdominoplasties are complex procedures that come with a variety of potential complications, which can significantly impact a patient's quality of life [2, 8, 25–29]. As such, patients often have numerous questions and concerns regarding both preoperative preparations and postoperative care. This underscores the importance of providing thorough and accessible information to address these concerns and ensure patients are well informed about their surgical journey [30, 31].

LLMs are trained on large amounts of data and fine-tuned to generate human-like text [32–35]. Consequently, they have the potential to significantly enhance the process of providing information to patients, serving as a valuable adjunct to traditional patient education methods [16, 36–41]. By delivering instant, accessible, and personalized responses to patient inquiries, LLMs can significantly enhance the patient care experience. These advanced tools are capable of tailoring information to the specific aftercare or preoperative education preferences of the surgeon, ensuring that patients receive responses that are directly relevant to their individual treatment plans. Moreover, LLMs can identify perioperative red flags, prompting the system to advise patients to immediately contact the appropriate clinician. In cases where patient questions indicate underlying concerns, LLMs could potentially facilitate immediate notification to the responsible surgeon, ensuring that critical issues are addressed promptly. Furthermore, the data captured from these interactions are invaluable, as they can be analyzed and shared with the treating team to improve the understanding of patients' perioperative needs. This approach not only reduces anxiety by clarifying doubts in real-time using empathetic vernacular but also improves overall patient satisfaction by creating a more responsive, informed, and patient-centered care environment. By summarizing patient concerns for surgeons, LLMs can streamline patient–surgeon communication by focusing on specific issues.

In aesthetic medicine, the need for lengthy explanations to repetitive patient inquiries highlights the inefficiencies of traditional interactions with surgeons and staff, emphasizing the importance of time optimization to prevent detracting from surgeons' core duties of surgery and consultation [42, 43]. In response, some practices have adopted strategies such as employing nurses for pre-consultation screening, reassurance, and procedural guidance. Incorporating LLMs into the preoperative phase presents a strategic innovation with the potential to significantly enhance patient education and screening processes. This technology can efficiently manage patient interactions, conserving valuable time for practitioners while maintaining high standards in patient care and support. This approach not only streamlines the workflow for surgeons, allowing them to focus on their primary duties without compromise, but also fosters a more informed and engaged patient population, ultimately contributing to improved perioperative outcomes and patient satisfaction.

This study compared the readability and reliability of responses from four leading LLMs—ChatGPT-3.5, Gemini, Claude, and Bing’s CoPilot—within the context of perioperative advice for abdominoplasty. Our findings highlight significant differences in the accessibility and quality of information provided by each LLM, with implications for their potential use in patient education and engagement.

The Flesch–Kincaid Grade Level and Flesch Reading Ease scores suggested that ChatGPT-3.5's outputs require the highest level of US education for comprehension, potentially limiting its accessibility to a broader patient demographic. Conversely, Bing’s CoPilot demonstrated the lowest required reading level, coupled with the highest patient-friendliness score, indicating its potential as a more universally accessible resource for patient education. The Coleman–Liau index presented a more nuanced view of readability, with CoPilot requiring the highest education level for comprehension, which contrasted with its performance in the Flesch–Kincaid analysis. This discrepancy underscores the complexity of assessing readability and the necessity of considering multiple metrics to gauge the accessibility of health-related information more accurately. More importantly, this situation highlights issues surrounding the accessibility of information. It has been observed that the health literacy levels among plastic surgery patients are typically insufficient [44–47]. Recommendations by the American Medical Association and the National Institutes of Health suggest that materials related to plastic surgery should be written at a sixth- to eighth-grade reading level [47]. However, recent research indicates that the readability of LLMs surpasses these recommendations, requiring a higher level of patient understanding [48, 49]. This discrepancy could potentially undermine the relationship between patients and healthcare providers, representing a significant barrier to the effective implementation of AI-driven chatbot perioperative tools in plastic surgery contexts.

Claude's superior DISCERN score indicates its reliability in providing information, suggesting that its outputs may be more suitable for patients seeking dependable advice. The consistency in advising professional consultation across all LLMs reinforces the importance of physician oversight in patient care. The responses provided by ChatGPT-3.5 exhibited a relatively comprehensive nature, encapsulating a substantial number of salient points presented in a manner conducive to facile comprehension. In contrast to ChatGPT-3.5's outputs for prompts 11 through 13, however, Claude was observed to furnish more expansive and specific delineations of the postoperative timeframe, thereby affording patients a more exhaustive preview of the trajectory of events post-op. Notably, Claude's avoidance of excessive medical jargon and clarity could make it particularly useful in facilitating patient understanding and engagement. CoPilot's use of emojis, colloquial language, and hyperlinks, unique among the LLMs evaluated, improved its approach towards patient engagement, potentially enhancing the user experience and comprehension through a more conversational and interactive format. However, its occasional lapses in providing expansive, specific medical advice such as in prompts 4, 8, and 12, failure to provide any advice in prompt 2, and the unexpected language switch in prompt 3 negatively affected its DISCERN score. This also accentuates the challenges of using LLMs in real-world settings, where accuracy and consistency are paramount as users may not be so patient as to re-prompt the AI model. Gemini’s emphasis on its non-professional status and conservative management strategies, while prudent, may limit the depth of information provided to patients, resulting in the trade-off between caution in advice and the richness of patient education content.

Despite the exciting prospects that LLMs provide, surgeons may be hesitant to integrate AI-driven perioperative tools into their practices due to the potential legal liability from errors in judgment or delays in care caused by such AI technologies. Such concerns are not unique to AI NLP interfaces, however. Similar concerns arise when training new or inexperienced clinical staff who may be triaging patient phone calls or messages [50, 51]. The risks of mistakes or delays exist whether it is an AI system or new human staff handling triage duties. Consequently, the legal liability worries are analogous regardless of whether AI NLP systems or newly hired personnel are being onboarded for perioperative assessment. At the moment, the surgeon should bear responsibility for all care and advice provided, regardless of whether it is given by a human or AI [52, 53].

It is imperative to acknowledge some limitations in our study, particularly the absence of actual patient involvement in evaluating the LLMs' outputs. Such engagements could provide invaluable insights into their practical utility, comprehensibility, and relevance of the responses generated by these LLMs, reflecting real-world needs and comprehension levels.

Additionally, the methodological design of our investigation is circumscribed by the participation of two plastic surgeons when curating the scores for the LLMs’ outputs. While their specialized acumen is critical for the appraisal of the AI models, this relatively small panel may impinge upon the external validity and generalizability of our conclusions. Expanding the group of evaluators to include more plastic surgery experts, and possibly other healthcare professionals, would provide a more comprehensive and balanced view of how effective these LLMs are in medical communication.

Furthermore, the focus of this study on perioperative inquiries to abdominoplasty delineated a specific segment of the expansive domain of plastic surgery. While this focus affords an in-depth examination within the context of abdominoplasties, it concurrently narrows the scope of inquiry, thereby restricting the comprehensive understanding of LLM applicability across multiple plastic surgical procedures. Consequently, there remains room for future research endeavors to investigate similar metrics in other plastic surgical procedures. This would not only augment the corpus of knowledge regarding LLM applications in healthcare but also guide the evolution of algorithmically sophisticated, procedure-specific models tailored to the intricate informational necessities of patients within the plastic surgery paradigm.

While no single LLM emerged as universally superior, each offered distinct advantages and challenges in readability, reliability, and patient engagement. These findings suggest that careful selection and possibly integration of multiple LLMs could optimize patient education and support. Future research should explore the direct impact of LLM-generated advice on patient outcomes and satisfaction, as well as investigate strategies for enhancing the accuracy, comprehensiveness, and personalization of LLM responses in clinical contexts.

The ethical integration of AI in surgical procedures raises significant concerns regarding privacy, consent, and human oversight [54, 55]. Ensuring AI systems like large language models adhere to healthcare privacy regulations, such as HIPAA, is crucial for protecting sensitive patient data. Patients must be fully informed about the role of AI, including how their data is used and potential risks, ensuring their consent is explicit and well documented. Moreover, maintaining human oversight is vital to ensure AI supplements rather than replaces professional medical judgment. This approach helps mitigate risks from AI inaccuracies and maintains the essential human element in healthcare, ensuring AI's role remains as an ethical adjunct to enhance patient care outcomes.

The study's limitations highlight the need for expanded research to validate AI applications in medical settings effectively. Notably, the absence of actual patient involvement restricts insights into the real-world utility and comprehensibility of AI communications. Future studies should include patient feedback to assess AI's effectiveness in clinical scenarios more accurately. Additionally, the evaluation based on only two plastic surgeons' perspectives may limit the findings' generalizability. Including a broader range of healthcare professionals as evaluators could provide a more comprehensive assessment. Furthermore, the study's focus on perioperative inquiries for abdominoplasty represents a narrow segment of plastic surgery. Broader research across various surgical procedures would help develop more precise AI models tailored to the diverse informational needs of different patient demographics, enhancing AI's clinical relevance and efficacy.

## Conclusion

This study highlighted significant differences between ChatGPT-3.5, Gemini, Claude, and Bing’s CoPilot, with each model showing unique strengths and weaknesses in terms of readability scores, DISCERN reliability, and patient-friendliness. The findings showcase the potential of LLMs to enhance patient education and support, but also emphasize the need for careful consideration of each model's accessibility and the quality of information provided. Future research should focus on integrating the advantages of these models to optimize patient education and engagement, while addressing the limitations identified to align more closely with the health literacy levels of the target patient demographics.
